# MUSIC CREATIVITY INTERVENTION FOR OLDER ADULTS WITH AND WITHOUT MCI: COGNITIVE BENEFITS AND NEURAL MECHANISMS

**DOI:** 10.1093/geroni/igad104.3709

**Published:** 2023-12-21

**Authors:** Christopher Fagundes, Lydia Wu-Chung, Anthony Brandt, Melia Bonomo, Bryan Denny, Jefferson Frazier, Karl Blench

**Affiliations:** Rice University, Houston, Texas, United States; Rice University, Houston, Texas, United States; Rice University, Houston, Texas, United States; Rice University, Houston, Texas, United States; Rice University, Houston, Texas, United States; Houston Methodist, Houston, Texas, United States; Musica, Houston, Texas, United States

## Abstract

Alzheimer’s and related dementias are debilitating conditions, and at this time, no cure has been identified. Older age is associated with cognitive decline and dementia. Developing and testing innovative non-pharmacological interventions to delay the loss of functional ability and understand the neurobiological mechanisms underlying the intervention’s efficacy is imperative. We conducted a semi-randomized clinical trial evaluating the effects of a novel music creativity curriculum on cognitive and neurological outcomes. Eighty older adults with and without a mild cognitive impairment (MCI) diagnosis were randomized to a 6-week music creativity intervention or a 6-week no-treatment control condition. Before and after the six weeks, both groups participated in a series of self-report and cognitive assessments; they also participated in resting-state fMRI scans to quantify whole-brain network patterns of modularity and flexibility. Those randomized to the music group exhibited improved overall cognitive function and working memory relative to the control group (assessed using standard clinical neuropsychological assessments). The association between being randomized to the music group and increased cognitive function was more robust among those who exhibited greater neural flexibility at baseline. The study shows that music creativity interventions may positively affect cognition, especially among those whose neural network structure is more dynamic or flexible. This study is innovative as it is among the first to identify possible neural mechanisms underlying why music creativity interventions confer a more significant cognitive benefit for some older adults than others.

